# Effect of 23-valent pneumococcal polysaccharide vaccine on medical expenses in Japan

**DOI:** 10.1186/1472-6963-14-S2-P108

**Published:** 2014-07-07

**Authors:** Kazunari Satomura, Toshitaka Nakahara, Suketaka Iwanaga, Megumi Noami, Keiko Kusaka, Kazuyoshi Harano

**Affiliations:** 1 Department of Public Health, Faculty of Medicine, Kyoto University, Kyoto, Japan

## Background

It has become popular to be vaccinated against pneumonia in Japan. It is reported that 23-valent pneumococcal polysaccharide vaccine prevents pneumonia and improves survival in nursing home residents in Japan [1]. However the effect on medical expenses of that vaccine in a local area has not yet been reported. We tried to evaluate it.

## Materials and methods

In a prefecture, which is located in western part of Japan, people over 75 years old have been assisted with being vaccinated by an association responsible for operation of the healthcare system for the elderly aged 75 and older since 2009. We tried to evaluate its effects on medical expenses.

## Results

Period A: From April 2009 till March 2012

Period B: From April 2012 till September 2012

The medical expenses for pneumonia of the vaccinated group (n=13689) and the non-vaccinated (n=126110) group are compared using the medical practitioners’ receipt for health insurance claim.

The medical examination rates in Period A were 0.403 per year in the vaccinated and 0.227 per year in the non-vaccinated. Those in period B were 0.549 in the vaccinated and 0.331 in the non-vaccinated. The increasing rate of the vaccinated seems to be suppressed compared to non-vaccinated.

Medical expenses in Period A were £365.2 in the vaccinated and £293.9 in the non-vaccinated. Those in Period B were £371.9 in the vaccinated and £172.1 in the non-vaccinated. The increase in medical expense seems to be suppressed by the vaccination.

## Conclusion

The rate of the vaccinated in this prefecture is small. And the Period B is too short. Also it seems that those that are vaccinated have some diseases or are compromised. However the vaccination seems to be effective in reducing medical examination rate of pneumonia and reducing the medical expenses for it.

These data have some limitations. Pneumonia on the medical practitioners’ receipt is not only caused by pneumococcus but also other causes such as virus. As the bills of medical treatment are issued every month, rates of diseases are sometimes overestimated. And the medical expenses for the inpatient was not calculated as they were not admitted only for pneumonia.

Effect of 23-valent pneumococcal polysaccharide vaccine seems to reduce pneumonia and medical expenses even in a local area.
